# Risk perception in peritoneal dialysis waste management: contributions for audiovisual material

**DOI:** 10.1590/0034-7167-2025-0498

**Published:** 2026-07-17

**Authors:** Gabriel Rodrigues Medeiros, Josué Laguardia, Silvia Regina Manfredi, Adriana Kelly Santos

**Affiliations:** I Instituto de Informação e Comunicação Tecnológica em Saúde. Rio de Janeiro, Rio de Janeiro, Brazil; II Fresenius Medical Care. Rio de Janeiro, Rio de Janeiro, Brazil; III Instituto Oswaldo Cruz. Rio de Janeiro, Rio de Janeiro, Brazil

**Keywords:** Peritoneal Dialysis, Medical Waste, Waste Management, Risk Communication, Audiovisual Aids., Diálisis Peritoneal, Residuos Sanitarios, Administración de Residuos, Comunicación de Riesgos, Recursos Audiovisuales.

## Abstract

**Objectives:**

to identify the risk perceptions of professionals, patients, and caregivers/family members regarding the proper disposal of waste from home peritoneal dialysis, with a view to verifying content/topics to be considered in the development of audiovisual material intended for patients and healthcare professionals.

**Methods:**

a qualitative study was conducted, including 17 semi-structured interviews with patients and caregivers, and an online focus group with eight experts in the fields of health, communication, and waste management, and one patient who is a radiologist undergoing peritoneal dialysis. Data were analyzed using thematic analysis.

**Results:**

doubts were reported regarding proper disposal, lack of standardized guidelines, and suggestions for selective collection and reverse logistics. Two categories emerged: “From care to waste”; and “Communication strategies of healthcare professionals”.

**Final Considerations:**

the importance of accessible educational strategies, with emphasis on risk communication and health literacy, to promote safe and sustainable practices in home care is highlighted.

## RESUMEN

**Objetivos:**

identificar las percepciones de riesgo de profesionales, pacientes y cuidadores/familiares con respecto a la correcta eliminación de los residuos de la diálisis peritoneal domiciliaria, para verificar el contenido/temas que deben considerarse en el desarrollo de material audiovisual destinado a pacientes y profesionales sanitarios.

**Métodos:**

se realizó un estudio cualitativo que incluyó 17 entrevistas semiestructuradas con pacientes y cuidadores, un grupo focal en línea con ocho expertos en salud, comunicación y gestión de residuos, y un paciente radiólogo en diálisis peritoneal. Los datos se analizaron mediante análisis temático.

**Resultados:**

se reportaron dudas sobre la eliminación adecuada de residuos, la falta de guías estandarizadas y sugerencias para la recolección selectiva y la logística inversa. Surgieron dos categorías: “De la atención al desecho”; y “Estrategias de comunicación de los profesionales de la salud”.

**Consideraciones Finales:**

se destaca la importancia de estrategias educativas accesibles, con énfasis en la comunicación de riesgos y la alfabetización en salud, para promover prácticas seguras y sostenibles en la atención domiciliaria.

## INTRODUCTION

Chronic kidney disease (CKD) is a progressive condition that is usually asymptomatic in its early stages, affecting more than 850 million people worldwide, with a global prevalence exceeding 10%^(1,2)^. In Brazil, it is estimated that between 3% and 6% of the adult population is affected^(3)^. Treatment is primarily performed using hemodialysis (HD), while peritoneal dialysis (PD), despite its advantages in terms of autonomy and lower cardiovascular burden, accounts for only 5.6% of cases, being limited by financial and structural obstacles and the reduced familiarity of healthcare professionals with this modality^(4)^.

Throughout the course of the disease and during renal replacement therapy, risk perception plays a significant role in treatment adherence and care safety. Studies indicate that caregivers and patients undergoing HD associate treatment with emotional overload and the occurrence of adverse events, such as hypotension and falls, reinforcing the feeling of vulnerability. Aspects such as stressful life events, negative perception of the disease, and difficulties in processing emotions also contribute to HD patients’ psychological distress^(5)^. In PD, the fear of infectious complications, especially peritonitis, intensifies the responsibility attributed to self-care, contributing to the perception of risk regarding the feasibility of home treatment^(6)^.

This understanding is influenced by subjective and contextual factors, such as prior experiences, personal values, and the technical and educational support offered by the nursing team, which includes practical guidance, doubt clarification, and patient autonomy encouragement. This is justified by the fact that home treatment requires not only technical skills, such as the correct handling of materials, but also a critical awareness of risks associated with preparing solutions, using supplies, and properly disposing of waste, in accordance with current health regulations^(7)^. Furthermore, repeating procedures can normalize behaviors, reducing vigilance and encouraging inappropriate practices with the potential to generate health hazards^(8)^.

The perception of risk by professionals, patients, and caregivers involved in home PD is a fundamental element in providing greater safety in care practices. Therefore, risk communication becomes an essential strategy in home nursing practice, and should occur in an empathetic, dialogical, and contextualized manner. More than just transmitting rules, nurses act as a mediator of risk perception, supporting patients in identifying dangers, making decisions, and developing safe and protective behaviors in home settings^(9)^. Although the use of technologies such as remote monitoring can promote autonomy and self-care, challenges remain regarding the reliability of practices performed at home, reinforcing the importance of continuous monitoring by the team and effective communication with patients^(10)^.

Despite the relevance of the topic, a significant gap is observed in the national and international literature regarding the understanding of the factors that influence risk perception and the management of waste generated by home PD. Specialized literature indicates that many patients have low levels of knowledge about the association between improper waste disposal and risks to public health or the environment, in addition to demonstrating inconsistent practices, especially in the handling of sharps^(11)^. Furthermore, studies have quantified that home PD generates tens of kilograms of plastic waste per patient per year^(12,13)^.

The scarcity of research addressing patients’ and caregivers’ perceptions, as well as the limited analysis of behavioral and cognitive factors influencing safe disposal, reinforces this scientific gap. Furthermore, the lack of consensus on effective educational strategies highlights the urgency of developing evidence-based instructional materials capable of promoting safe and sustainable practices in home settings^(14,15)^. In this regard, studies indicate the central role of nursing in promoting health literacy (HL), providing ongoing support to patients, and monitoring safe and environmentally responsible practices^(16-18)^.

Thus, this article offers pathways for understanding the elements that affect risk perception and waste management among users and caregivers involved in home PD. Furthermore, it contributes to the development of educational and communicative actions focused on patient safety and environmental sustainability.

## OBJECTIVES

To identify the risk perceptions of professionals, patients, and caregivers/family members regarding the proper disposal of household PD, with a view to verifying content/topics to be considered in the development of audiovisual material intended for patients and healthcare professionals.

## METHODS

### Ethical aspects

The study was conducted in accordance with national and international ethical guidelines, and was approved by the *Escola Politécnica de Saúde Joaquim Venâncio* Research Ethics Committee, whose opinion is attached to this submission. All participants were informed about the objectives, risks, and benefits of the research, as well as their freedom to withdraw without prejudice to their treatment. The Informed Consent Form was signed in person and/or completed via electronic form (Google Forms^®^), with a copy sent to participants. Consent was verbally reaffirmed at the beginning of the application of each research technique. Confidentiality was ensured through the use of pseudonyms in the presentation of the study results.

### Study design

This is a qualitative, exploratory study, guided by Consolidated Criteria for Reporting Qualitative Research guidelines.

### Methodological procedures

The methodological procedures focused on patients’, caregivers’, and professionals’ experiences and perceptions regarding waste disposal and risk communication in the context of home PD. Semi-structured interviews and online focus groups (FGs) were used as data collection techniques.

### Study setting

The study was conducted at a private healthcare unit affiliated with the Brazilian Health System (In Portuguese, *Sistema Único de Saúde* - SUS), located in Nova Iguaçu, Baixada Fluminense, Rio de Janeiro, which monitors approximately 350 patients undergoing renal replacement therapy, being the only one in the region to offer PD. However, this treatment is not available through the SUS due to the low reimbursement value, being performed exclusively privately. This unit was chosen due to its importance in the referral and counter-referral flow at the regional and municipal levels, the number of patients served, and the first author’s professional involvement in the clinic’s nursing team.

### Data source

Participant selection was carried out by non-probabilistic convenience sampling, recruiting two groups. The inclusion criteria for the first group were a minimum age of 18 years, a minimum of three months on home PD and/or in the follow-up of PD patients, and preserved clinical and cognitive conditions. In the first group, of the 30 patients eligible to participate in the study, 13 were excluded for the following reasons: seven because they were undergoing training for the use of PD; three because they missed their appointments; two because they were hospitalized; and one due to the absence of the responsible caregiver. In addition to the patients, this first group also included ten caregivers, as they were directly responsible for carrying out the therapy at home, after having been previously trained by the nursing team.

The second group comprised eight invited participants, via email and WhatsApp^®^, to form the online FG. Participants were selected by convenience and through the authors’ network of contacts. The sample composition considered engagement with the study topic. Thus, professionals with experience in the following areas were invited: nephrology (nurse); waste management (architect and nurse); HL (physician and social worker); and communication (filmmaker, risk communication specialist, and journalist/radio broadcaster, the latter also being a patient with PD).

### Data collection and organization

The first group participated in semi-structured interviews, conducted in July 2024, at the clinic facilities, in a private room and at a time chosen by the interviewees. The interviews were conducted by the first author, with authorized recording and subsequent transcription. The interviews aimed to identify risk perceptions related to home PD waste disposal, based on the experiences of patients and caregivers directly involved in the daily practice of treatment. The choice of a qualitative approach was justified by its ability to capture, in rich detail, the meanings attributed by participants to their experiences^(19,20)^.

The interviews were conducted following a script with open-ended questions previously prepared by the first two authors, from which personal data were identified (name, work/occupation status, education/training, age, marital status), knowledge about the disease and its treatment with PD (time of diagnosis, home care procedures), and perception of waste generated by home PD and the risks related to it. The script was adapted to the dynamics of each interview, totaling 102.27 minutes of recording (an average of 6.04 minutes per session).

Indeed, through dialogue between researcher and participant, it was possible to access subjective and contextual dimensions of home care, revealing knowledge, doubts, empirical strategies, and informational gaps that would be difficult to uncover using quantitative methods^(21,22)^. More than just objective data, the interviews allowed for an understanding of how subjects interpret risks, manage waste, and what they consider essential to communicate in educational material. Therefore, the data obtained in this stage supported the FG script development, as well as the topics contained in the script of the audiovisual material on home PD waste disposal. Thus, the FG was configured as the next stage of investigation, aimed at validating and collectively deepening these findings, enabling the comparison of perceptions and the shared construction of the educational material.

The FG was scheduled using the Doodle^®^ tool, and the meeting took place in November 2024 via the Microsoft Teams^®^ platform, lasting 171 minutes. The FG was coordinated by the first and fourth authors, who observed the interactions among participants and moderated the debate, in accordance with the proposal described by Alves for online FGs^(23)^. Initially, the coordinator led the presentations and explained the FG’s objective, which was to create a script with relevant content for the production of audiovisual material about safe disposal of waste generated by home PD. Subsequently, using a script with open-ended questions, previously prepared by the authors, knowledge and perceptions related to CKD, PD home treatment, and the waste generated in this type of treatment, as well as associated risks, were discussed (Chart 1).

**Chart 1:** Focus group with experts and patient: dynamics and questions, Rio de Janeiro, Brazil, 2025

1^st^ moment - Presentation and general information about the focus group				

	Presentation of ethical issues		Informed Consent Form	

	Guidelines on the focus group rules		It will be led by three mediators; It will be recorded in video and audio, while preserving participants’ identity; It will last an average of 90 to 140 minutes, with ten-minute breaks; The discussion will begin with open-ended questions related to the study’s topic; Discussions can be conducted via text or audio, at the discretion of each participant; Participants will be able to insert emoticons to signal expressions, feelings, and emotions experienced, as well as at the moderator’s request. Before concluding the discussion of each question, participants will be invited to insert emoticons representing their expression regarding the topic discussed.	

**2^nd^ moment - Debate**				

	Questions about participants’ experiences with chronic kidney disease and peritoneal dialysis		What is your level of knowledge about chronic kidney disease? Do you have any experience with peritoneal dialysis? Do you know how it is done?	

	Questions about peritoneal dialysis waste disposal		Do you know what types of waste are generated? Have you ever wondered about the proper disposal of home peritoneal dialysis waste? How can we dispose of this waste safely?	

	Scriptwriting for audiovisual media		Presentation and analysis of existing instructional materials for patients and families undergoing peritoneal dialysis; What do we need to communicate the safe disposal of peritoneal dialysis waste to patients, families, and caregivers? Is audiovisual the most appropriate means of dissemination? What information can be included? What difficulties exist? What solutions can we find?	


### Data analysis

The analysis of interviews and the FG was conducted using a mixed-methods approach, with descriptive quantitative analysis of sociodemographic variables and qualitative analysis based on thematic content analysis techniques^(24)^. Content analysis was developed in three stages. In the first stage, pre-analysis, full transcripts of interviews and the FG were made, and a preliminary reading of the transcribed information was conducted, identifying the information, the frequency with which words are repeated, and the initial topics. Each text was observed in isolation and transversally, listing the elements of meaning based on the initial topics. In this stage, initial topics were selected that gave rise to thematic subgroups. Some of these topics emerged unexpectedly from participants’ statements, while others derived directly from the questions formulated in the interview scripts and the FG.

The second stage, material exploration, involved the coding process of the initial topics, taking into account the selection of words, phrases, and paragraphs that generated the recording units, as well as the rules defined for counting, classifying, and grouping information into thematic categories, based on semantic convergence^(24,25)^. Thus, the texts from the interviews and the FG were grouped into initial topics, recording units, and subsequently grouped into thematic units.

In the third stage, inference and interpretation, the results from the coding stage were processed, taking into account the analysis of the speeches based on exhaustiveness, representativeness, homogeneity, and relevance criteria. This procedure aimed to identify the expressed and latent content present in the analyzed material. Subsequently, these data were regrouped through analytical categories and their respective subcategories, allowing for consistent interpretations in relation to the object of study. The statements cited in the results are identified by the letters “P” (patient) and “C” (caregiver), followed by the insertion number, sex, and age (P:01, female, 30 years old). The radio broadcaster patient, who also participated in the FG, was identified by the initials “RP”. Specialists were identified by the letter “S”, followed by the initials of their area of expertise, such as NS (nephrology), HLS (health literacy), EHS (environmental health), WMS (waste management), RCS (risk communication), and FS (filmmaker).

## RESULTS

Seventeen people (seven patients and ten caregivers) directly involved in home PD participated in the interviews, ranging in age from 18 to 89 years. Most patients were over 40 years old, married, and had education levels ranging from elementary to complete higher education. Among the caregivers, there was a predominance of females, aged between 40 and 69 years, with higher levels of education and employment. Regarding treatment, approximately two-thirds of patients used automated PD, and most had been in therapy for less than three years. Most patients and caregivers resided in municipalities of the Baixada Fluminense and the Metropolitan Region of Rio de Janeiro (Table 1).

**Table 1:** Sociodemographic and clinical characteristics according to participant type (patient or caregiver), Rio de Janeiro, Brazil, 2025

Variable	Patient		Caregiver	
	n	%	n	%
**Sex**				
Male	3	42.9%	**4**	**40%**
Female	4	57.1%	6	60%
**Age range (years)**				
18-29	2	28.6%	-	-
30-39	-	-	2	20%
40-69	4	57.1%	7	70%
70-89	1	14.3%	1	10%
**Marital status**				
Single	1	14.3%	1	10%
Married	5	71.4%	9	90%
Common-law marriage	-	-	-	-
Divorced	1	14.3%	-	-
Widowed	-	-	-	-
**Education**				
Illiterate	-	-	-	-
Incomplete elementary school	1	14.3%	-	-
Complete elementary school	1	14.3%	1	10%
Incomplete high school	1	14.3%	-	-
Complete high school	2	28.6%	4	40%
Incomplete higher education	-	-	2	20%
Complete higher education	2	28.6%	3	30%
**Professional activity**				
Student	-	-	-	-
Education	1	14.3%	1	10%
Industry/commerce	1	14.3%	2	20%
Informal work	1	14.3%	2	20%
Freelancer	-	-	2	20%
Domestic work	2	28.6%	2	20%
Retired	1	14.3%	-	-
Civil servant	-	-	-	-
No activity	1	14.3%	2	20%
**Dialysis modality**				
Automated peritoneal dialysis	11	64.7%	-	-
Continuous ambulatory peritoneal dialysis	6	35.3%	-	-
**Treatment time (years)**				
**Up to three years**	14	82.4%	-	-
> three and < five years	2	11.8%	-	-
> five years	1	5.9%	-	-

The online FG included one patient and seven professionals with degrees in various fields (nursing, medicine, social work, architecture, communication, and audiovisual production), five of whom held doctoral degrees. There was a predominance of female participants, with the majority residing in the state of Rio de Janeiro.

## DISCUSSION

In the content analysis of the interviews and the FG, described in Chart 2, 37 initial topics were identified, regrouped into four subcategories: a) Experiences and knowledge about chronic kidney disease and peritoneal dialysis; b) Practices and care related to home peritoneal dialysis; c) Waste generated in home peritoneal dialysis: safe disposal; d) Communication and health: audiovisual material about home peritoneal dialysis. Finally, these four subcategories were regrouped into two categories of analysis, which synthesize the interpretations and inferences in light of specialized literature, namely “From care to waste” and “Communication strategy used by healthcare professionals in peritoneal dialysis”.

**Chart 2:** Categories, subcategories, and main inferences from thematic analysis, Rio de Janeiro, Brazil, 2025

Category	Subcategory	Initial topics
**From care to waste**	Experiences and knowledge about chronic kidney disease and peritoneal dialysis	- Chronic kidney disease: experience, knowledge, and feelings - Peritoneal dialysis: experience, feelings, myths, and truths - Social vulnerability of people undergoing peritoneal dialysis in the Brazilian Health System - Sociocultural aspects related to peritoneal dialysis (impact on social life, daily routine, acceptance, and adaptation to treatment) - Family support network in coping with the disease
	Practices and care related to home peritoneal dialysis	- Role of nurses in patient/family/caregiver training - Peritoneal dialysis technique/preparation of materials - Awareness of performing the correct technique - Ease of application - Rigorous hygiene - Materials used in peritoneal dialysis
	Waste generated in home peritoneal dialysis: safe disposal	- Types of waste generated by peritoneal dialysis - Misinformation about home peritoneal dialysis waste disposal - Insecurity in handling waste generated by peritoneal dialysis - Concern for workers who handle the discarded waste - Reflection on social inequalities in each region of Brazil in relation to waste disposal - Public policies and waste - Household waste is inconvenient - Waste management in home settings - Benefits of proper disposal for people and the environment - Convenient disposal - Short-term solution: collection of materials by the transport company - Medium and long-term solution: reverse logistics.
**Communication strategy used by healthcare professionals in peritoneal dialysis**	Communication and health: audiovisual material about home peritoneal dialysis	- Communication between healthcare professionals and patients/caregivers - Use of simple language - Salutogenesis perspective - Communication strategies: setting the scene; turning point; positive messages; showing empathy in the material; key messages; target audience; channels and formats; approaches to be used - Realistic characters: nurse and patient in the script

**Chart 3:** Proposal for an audiovisual script, Rio de Janeiro, Brazil, 2025

Title: “Peritoneal dialysis: caring for oneself and the world”
**TARGET AUDIENCE:** Patients undergoing peritoneal dialysis. Family members and formal and informal caregivers. Healthcare professionals working in home health education.
**ACTION AND PARTICIPANTS** Main format: realistic conversation between nurse and patient, simulating a home guidance session. Setting: adapted home environment or welcoming care space. Participants: Nurse: empathetic, clear, careful posture. Patient: represents common doubts and feelings. (Optional) Family member or caregiver present as support and active listener.
**COMMUNICATION STRATEGIES** Simple and direct language. Use of health literacy concepts - visual explanations, simple comparisons, and practical examples. Risk communication (e.g., infection risks, improper disposal). Salutogenic perspective: focus on autonomy, well-being, and aspects of patients’ and caregivers’ daily lives. Emphasis on environmental aspects: ecological awareness in waste disposal.
**FORMAT** **Option 1 - Single video** Duration: approximately ten minutes. Compact format, ideal for presentation in outpatient clinics, intake sessions, or as standalone digital content. **Option 2 - Series (educational miniseries)** Three episodes with a total duration of approximately ten minutes. Allows for gradual in-depth exploration of topics, a stronger connection with the characters, and reinforcement of learning. It can be used serially or independently, serving different purposes and audiences.

### From care to waste

The “From care to waste” category comprises three subcategories that address the experience of becoming ill, the daily routine of home treatment for PD, and the challenges related to care, waste management, and risks associated with managing the waste generated.

The first subcategory, “Experiences and knowledge about chronic kidney disease and peritoneal dialysis”, highlights the late discovery and limited or lack of knowledge about kidney disease and PD. Reports from patients and caregivers indicate that CKD diagnosis often occurred unexpectedly, after severe episodes of illness. The experience of becoming ill is unique, and the start of therapy is experienced as a moment of symbolic loss and grief.

*I put my life on hold because of dialysis.* (P1, male, 23 years old)

*You have an illness that limits you, right? You no longer have the life you once had, right? The life of being able to have total freedom in every aspect of life.* (P8, male, 49 years old)

The emotional burden and significance of the illness impact the body, generate psychological problems, and impose dependence on specialized care and changes in family and social routines. The decision to seek therapy proved more viable with professional and family support, although PD is still not widely known.

*In the beginning, I was disciplined, I did everything perfectly. And then, I kind of let myself go. That’s also a psychological problem* [...] *we deprive ourselves too much in order to live.* (P14, female, 29 years)

*The nurse presented me with this new possibility... that already gave me a certain sense of reassurance.* (RP)

*I have the help of my father, which is very important* […] *I think it’s 50% of the treatment.* (RP)

Difficulty accessing PD and misinformation contribute to myths about eligibility criteria, such as rigid requirements regarding domicile.

*When I first learned about this treatment, people told me I should have a hospital room at home.* (RP)

*There used to be a concept that, to undergo peritoneal dialysis, you had to have a private room, no dogs, and a separate bathroom. This concept has changed, partly because peritoneal dialysis serves people who live far away.* (NS)

The “Practices and care related to home peritoneal dialysis” subcategory highlights the importance of training conducted by nurses in preparing the patient, family, and caregiver, promoting the acquisition of required techniques and safety in home management. Participants reported that the routine includes rigorous hygiene care, environment preparation, and correct supply handling.

*First, we have to clean the whole environment, right? The room. I clean everything. I wipe everything down with alcohol and bleach. I wait 30 minutes. Then I wash the devices with soap, wash my hands thoroughly, wipe them down with alcohol, and plug them back in. After plugging them in, I drain the liquid, right? And I’m done. And I go back to my normal life”.* (P17, female, 52 years old)

One aspect that caught patients’ and caregivers’ attention is the volume of supplies delivered monthly to their homes, packaged in cardboard boxes protected by plastic wrapping. Furthermore, regarding treatment, the need to use materials for hygiene and safety during the procedure was highlighted.

*The boxes containing peritosteril, which is the liquid we use in peritoneal dialysis, and boxes with the tubing* [...] *which is how we connect to the machine, the bag, and my catheter, arrived at my house. And then, for each dialysis session, I use one package.* (RP)

*There’s 70% alcohol for hand sanitization, and masks to prevent and minimize any risk of infection. The dressing, the hygiene, using saline solution, gauze* [...] *the issue of the needle is somewhat relative, because sometimes some fibrin appears that ends up blocking the passage of liquid in the catheter, and then we inject a solution into the bag, but this needle procedure doesn’t necessarily have to be done every day.* (RP)

The third subcategory, “Waste generated in home peritoneal dialysis: safe disposal”, reveals information gaps, insecurity in waste management, and concern about risks to public health and the environment. Although some describe their disposal routines, many are unclear about the proper procedure.

*I take the material, right? I cut off that liquid... I throw the liquid away in the tank* [...] *so it can go to the sewer* [...] *and I throw away the material.* (P17, female, 52 years old)

*Then, after it’s finished, we also put on our masks, remove them from the equipment, disinfect everything with alcohol, and discard the material.* (C16, female, 35 years old)

There were reports expressing uncertainty about the risks and a recurring concern about the exposure of waste collection workers.

*So, we, you know* [...] *put it in a separate bag for the garbage collector to take away* [...] *because we’re afraid of contamination.* (P17, female, 52 years old)

*The risk is not just for the patient; it’s for everyone involved.* (C2, female, 43 years old)

The statements reinforce a lack of information about this stage of treatment. This is aggravated by the diversity of materials used, which makes categorization according to risk and, consequently, proper disposal difficult.

*There’s a lack of information when it comes to the disposal process. There isn’t a very specific manual yet. That plastic bag containing liquid waste, the tubing from the IV set, the catheter cap-are you going to throw it in the trash? Shouldn’t there be proper preparation?* (RP)

Sharps disposal and waste accumulation pose a challenge that also raises questions.

*Yes, that needle, that’s exactly what we were wondering about. I collect these needles and take them to the nurse at the clinic to dispose of them.* (RP)

*I have hundreds of boxes that I can’t dispose of because the garbage collector doesn’t want to take them, and burning them is very difficult, nobody wants them. They’re not being sold anymore, nobody wants them because they’re cheap, but then they become a burden.* (C13, male, 58 years old)

Experts pointed out flaws in the legislation, highlighting that, in the domestic environment, waste can generate risks and nuisances, and, in places without adequate sanitary infrastructure, these risks become even more serious.

*We have very well-established environmental legislation, but there are still flaws in this issue of waste disposal* [...] *and within this issue, we have the classification of these wastes.* (EHS)

*Waste is a matter of scale; the larger the scale, the bigger the problem. In the domestic environment, it poses risks or inconveniences: odor, attraction of vectors* [...] *the relationship with the waste determines what one will do with it.* (WMS)

*Today we have two companies in Brazil that supply peritoneal dialysis equipment: one discharges the effluent directly into the sewer, the other does not. They use bags where the effluent is stored and then drained normally into the toilet.* (NS)

Improper disposal of wastewater in unsanitary locations, such as septic tanks near wells, increases the risk of contamination.

*We have situations where patients use well water and dispose of the sewage in a septic tank that is 10 or 15 meters away from that well* [...] *when they dump that into a septic tank and draw water from the water table, they may be sabotaging themselves.* (WMS)

As solutions, experts highlighted reverse logistics and the collection of materials by the carrier, as well as the use of rigid containers for sharps.

*Legally, the generator is also responsible for the treatment. The manufacturer will have to collect it somehow.* (WMS)

*Whoever delivers the material could collect cardboard and recyclable plastic, not the bags containing dialysis waste.* (NS)

*In the medium and long term, implement reverse logistics. In the short term, create micro-logistics where the delivery person periodically collects specific materials.* (WMS)

*I recommend collecting needles and syringes in wide, rigid containers so that they can be disposed of as correctly as possible.* (EHS)

*In most municipalities, there is no separate waste collection system.* (WMS)

### Communication strategy used by healthcare professionals in peritoneal dialysis

The “Communication strategy used by healthcare professionals in peritoneal dialysis” category, derived from the “Communication and health: audiovisual material about home peritoneal dialysis” subcategory, brings together reflections from different professionals and patients, highlighting fundamental elements to support the development of the script for the educational audiovisual material on PD waste disposal.

Participants endorsed the audiovisual format, indicating its potential for interactivity, and recommended the inclusion of “QR Codes and other links with hierarchically organized information” (RCS). They also proposed short, objective videos with direct and motivational language, according to the audience profile and the dissemination channel. Focusing on HL, the importance of prioritizing content to depict the daily routine of treatment was discussed - receiving supplies, preparing for the PD procedure, managing waste, and safe disposal. To this end, participants suggested that the characters should be patients, professionals, and caregivers involved in care, emphasizing humanization of care, autonomy, patient safety, and quality of life. When composing this narrative, it was also recommended to address the interaction between healthcare professional, patient, and caregiver, emphasizing the bond, active listening, and empathy. According to them, communication is a two-way street, promoting co-responsibility in care, because:

*It’s not enough to just say what’s right. You have to listen, understand the difficulties.* (FS)

*First, you need to speak in a language that the audience will understand. There’s no point in speaking medical jargon.* (RP)

In line with this perspective, risk communication was identified as strategic for patient safety, with the construction of an accessible narrative that motivates action being indicated, associating proper disposal with individual and collective well-being. Participants removed the need to contextualize waste disposal within the complete therapy process, considering that “it is impossible to understand disposal without understanding the entire process that precedes it” (FS).

Based on these elements, a preliminary script for an audiovisual presentation on home PD and insurance claim disposal is proposed, focusing on health promotion and empowerment of the individuals involved. The preliminary script addresses the following aspects: setting the scene; defining key messages; conceptual clarity; using motivational narratives; adapting the format to the broadcast channel; encouraging self-care; and valuing the bond between professional and patient. Chart 3 describes these aspects.

## DISCUSSION

The lack of knowledge about the factors that lead to CKD is widely documented^(26)^. Since it is asymptomatic and slow-progressing, it is usually diagnosed late, when dialysis is already necessary^(27)^. The lack of information, possibly resulting from insufficient communication from healthcare professionals, affects different population groups unequally. The scarcity of studies on the impact of media and providers on understanding the disease hinders the definition of more effective communication strategies^(28)^. CKD impacts patients’ lives, frequently leading to job loss and reduced social activity. The progression of the disease causes physical and psychosocial decline, requiring continuous monitoring, compromising autonomy, and increasing dependence on others^(29,30)^. Treatment demands disrupt life plans, generating feelings of limitation and loss of control^(30)^.

PD emerges as an alternative that promotes autonomy through home treatment, although it is underutilized due to structural and financial limitations, as well as a lack of patient knowledge, exacerbated by inadequate information provided by healthcare teams^(26)^. In this context, nurses and nephrologists play a central role in guiding patients, encouraging participation in decisions, and promoting adherence to treatment^(31)^.

In Brazil, a country marked by regional, cultural, and socioeconomic inequalities, people living in homes without adequate conditions for home therapies such as PD become more vulnerable. The lack of basic infrastructure, coupled with low levels of education and family income, compromises the effectiveness of treatment and increases the risk of complications, including those related to the improper management of healthcare waste at home^(32)^. Family support is fundamental to coping with CKD demands, providing emotional, social, and financial support, promoting adherence and quality of life^(33)^.

PD’s success depends on a suitable environment, ongoing training, and regular follow-up. The nurse leads the training of patients, family members, and caregivers, ensuring technical proficiency, correct execution of the procedure, and autonomy in home management. Furthermore, it provides guidance on the safe and responsible handling of generated waste^(34)^. However, PD generates various types of plastic waste, such as polyvinyl chloride and polypropylene, sharps, glass ampoules, dialysis solutions, and paper/cardboard, in significant annual volumes^(13)^. The adoption of sustainable strategies by service providers and healthcare facilities, such as recycling, reverse logistics, and “Green Nephrology” practices, can reduce their environmental footprint by promoting water reuse and the use of recyclable materials^(12,35)^.

Improper waste management poses occupational, environmental, and public health risks. Sharps and ampoules can cause accidents, and the incorrect disposal of medications contributes to bacterial resistance^(36,37)^. Furthermore, inadequate hygiene techniques during PD increase the risk of peritonitis and premature transfer to HD^(38,39)^.

In Brazil, household waste disposal practices reflect regional and socioeconomic inequalities, with improper disposal of syringes, needles, and plastics being frequent^(32)^. Despite the regulations of Collegiate Board Resolution 222 of March 28, 2018 from the Brazilian National Health Regulatory Agency, many waste materials are still disposed of in regular trash, increasing health and environmental risks^(32,40)^.

Supplies should be stored in dry, clean locations and inspected for packaging integrity^(41)^. Aseptic technique, with emphasis on hand hygiene, remains an essential preventive measure^(38,39)^. Reverse logistics and recycling are promising strategies, although they face technical and financial challenges, while incineration, despite its efficiency, requires high environmental control and investment^(12,36)^.

Communication between professionals, patients, and caregivers is essential for understanding, adherence, and well-being. Simple, direct, and accessible language increases efficiency, reduces ambiguity, and strengthens safety^(42)^. The salutogenic perspective guides communication, emphasizing factors that promote health and strengthen a sense of coherence, improving the ability to cope with adversity^(43)^.

Structured communication strategies, including contextualization of the topic, key messages, practical examples, positive reinforcement, and the inclusion of realistic characters such as nurses and patients, facilitate audience identification and promote greater engagement and understanding^(42)^. Digital channels and formats, aligned with the target audience and institutional values, enhance effectiveness^(44)^. Continuous training and specializations enhance skills, job satisfaction, and clinical outcomes^(45)^.

In summary, integrating salutogenesis with planned communication strategies strengthens autonomy and promotes humanized care, safety, and well-being, serving as a basis for accessible, welcoming, and informative educational materials geared towards daily practice, risk prevention, and environmental care.

### Study limitations

Convenience sampling stands out among the main limitations of this study, which may have introduced selection bias and restricted the representativeness of the different profiles of patients and caregivers in PD, and the online FG, which may have limited the observation of non-verbal elements, such as facial expressions and gestures, important for qualitative analysis. Furthermore, although a preliminary script for educational audiovisual material was developed, the final video product will still be developed and validated with the chosen audience.

### Contributions to nursing, health, or public policy

This study reinforces the relevance of nursing’s role in the care of PD patients and in health education, especially given the need to ground risk perception in technical knowledge, developing HL among patients and caregivers. The results of this study aimed to support the production of a script for a contextualized audiovisual presentation that contributes to improving the performance of nurses, patients, and caregivers involved in promoting safe and environmentally responsible home care practices.

## FINAL CONSIDERATIONS

The study achieved its objective by identifying key aspects of risk perception in home PD waste disposal, based on reports from patients, caregivers, and professionals. The results highlighted doubts about proper disposal, gaps in Brazilian legislation, and the relevance of environmentally safe practices such as selective collection and reverse logistics.

Participants’ contributions highlighted the need for accessible educational strategies, emphasizing risk communication and HL as tools to promote autonomy, critical thinking, and safe practices. The preliminary script for educational audiovisual material, developed from this information, emerges as a concrete response to the identified gaps, with the potential to support home care, train professionals, and broaden the discussion on sustainability in renal therapy.

## Data Availability

The research data are available only upon request.
