# Supplementary material for: Risk perception in peritoneal dialysis waste management: contributions for audiovisual material
Source: Rev Bras Enferm. 2026 Jul 17;79:e20250498. doi: 10.1590/0034-7167-2025-0498 (PMC13379241; doi:10.1590/0034-7167-2025-0498)
Supplement: Translation

# Translation: Percepção de risco no manejo de resíduos da diálise peritoneal: subsídios para um material audiovisual

**DOI:** 10.1590/0034-7167-2025-0498pt

**Authors:** Gabriel Rodrigues Medeiros, Josué Laguardia, Silvia Regina Manfredi, Adriana Kelly Santos

**Affiliations:** I Instituto de Informação e Comunicação Tecnológica em Saúde. Rio de Janeiro, Rio de Janeiro, Brasil; II Fresenius Medical Care. Rio de Janeiro, Rio de Janeiro, Brasil; III Instituto Oswaldo Cruz. Rio de Janeiro, Rio de Janeiro, Brasil

**Keywords:** Diálise Peritoneal, Resíduos de Serviços de Saúde, Gerenciamento de Resíduos, Comunicação de Risco, Recursos Audiovisuais.

## Abstract

**Objetivos:**

identificar as percepções de risco de profissionais, pacientes e cuidadores/familiares sobre o descarte adequado dos resíduos da diálise peritoneal domiciliar, com vistas à verificação de conteúdos/temas a serem considerados na elaboração de material audiovisual destinado a pacientes e profissionais de saúde.

**Métodos:**

estudo qualitativo, com 17 entrevistas semiestruturadas com pacientes e cuidadores, e grupo focal *online* com oito especialistas das áreas da saúde, comunicação e gestão de resíduos, e um paciente radialista em diálise peritoneal. Os dados foram analisados pela técnica de análise temática.

**Resultados:**

foram relatadas dúvidas quanto ao descarte adequado, ausência de orientações padronizadas e sugestões de coleta seletiva e logística reversa. Emergiram duas categorias: “Do cuidado ao resíduo”; e “Estratégias de comunicação dos profissionais de saúde”.

**Considerações Finais:**

destaca-se a importância de estratégias educativas acessíveis, com ênfase na comunicação de risco e na literacia para a saúde, para promover práticas seguras e sustentáveis no cuidado domiciliar.

## INTRODUÇÃO

A doença renal crônica (DRC) é uma condição progressiva e geralmente assintomática nos estágios iniciais, afetando mais de 850 milhões de pessoas no mundo, com prevalência global superior a 10%^(1,2)^. No Brasil, estima-se que entre 3% e 6% da população adulta seja acometida^(3)^. O tratamento é majoritariamente realizado pela hemodiálise (HD), enquanto a diálise peritoneal (DP), apesar de suas vantagens em termos de autonomia e menor sobrecarga cardiovascular, representa apenas 5,6% dos casos, sendo limitada por entraves financeiros, estruturais e pela reduzida familiaridade dos profissionais de saúde com essa modalidade^(4)^.

No decorrer da doença, e durante a terapia renal substitutiva, a percepção de risco exerce papel relevante na adesão ao tratamento e na segurança do cuidado. Estudos apontam que cuidadores e pacientes submetidos à HD associam o tratamento à sobrecarga emocional e à ocorrência de eventos adversos, como hipotensão e quedas, o que reforça a sensação de vulnerabilidade. Aspectos como eventos estressantes da vida, percepção negativa da doença e dificuldades em processar emoções também contribuem para o sofrimento psicológico de pacientes em HD^(5)^. Na DP, o medo de complicações infecciosas, especialmente da peritonite, intensifica a responsabilidade atribuída ao autocuidado, contribuindo para a percepção de risco quanto à viabilidade do tratamento domiciliar^(6)^.

Esse entendimento é atravessado por fatores subjetivos e contextuais, como experiências prévias, valores pessoais, e suporte técnico e educativo oferecido pela equipe de enfermagem, que abrange orientações práticas, esclarecimento de dúvidas e estímulo à autonomia do paciente. Isso se justifica pelo fato de que o tratamento em casa exige não apenas habilidades técnicas, como a correta manipulação dos materiais, mas também uma consciência crítica sobre os riscos associados ao preparo das soluções, ao uso de insumos e ao descarte adequado dos resíduos, em conformidade com a legislação sanitária vigente^(7)^. Aliado a isso, a repetição dos procedimentos pode naturalizar comportamentos, reduzindo a vigilância e favorecendo práticas inadequadas com potencial de gerar perigos sanitários^(8)^.

A percepção de risco por parte de profissionais, pacientes e cuidadores envolvidos na DP domiciliar é um elemento fundamental para prover maior segurança nas práticas de cuidado. A comunicação de risco, portanto, torna-se uma estratégia essencial na prática de enfermagem domiciliar, devendo ocorrer de forma empática, dialógica e situada. Mais do que transmitir normas, o enfermeiro atua como mediador da percepção de risco, apoiando o paciente na identificação de perigos, na tomada de decisão, e na construção de condutas seguras e protetoras no ambiente domiciliar^(9)^. Ainda que o uso de tecnologias como o monitoramento remoto possa favorecer a autonomia e o autocuidado, persistem desafios quanto à confiabilidade das práticas realizadas no domicílio, reforçando a importância do acompanhamento contínuo por parte da equipe e da comunicação efetiva com o paciente^(10)^.

Apesar da relevância do tema, observa-se uma lacuna significativa na literatura nacional e internacional quanto à compreensão dos fatores que influenciam a percepção de risco e a gestão dos resíduos gerados pela DP domiciliar. A literatura especializada indica que muitos pacientes apresentam baixo conhecimento sobre a associação entre o descarte inadequado de resíduos e os riscos à saúde pública ou ao meio ambiente, além de evidenciarem práticas inconsistentes, especialmente no manejo de materiais perfurocortantes^(11)^. Além disso, estudos quantificaram que a DP domiciliar gera dezenas de quilos de resíduos plásticos por paciente ao ano^(12,13)^.

A escassez de investigações que abordem a percepção dos pacientes e cuidadores, assim como a limitada análise dos fatores comportamentais e cognitivos que influenciam o descarte seguro, reforça essa lacuna científica. Além disso, a ausência de consenso sobre estratégias educativas eficazes evidencia a urgência de desenvolver materiais instrucionais fundamentados em evidências, capazes de promover práticas seguras e sustentáveis no contexto domiciliar^(14,15)^. Nesse quesito, estudos indicam a centralidade do papel na enfermagem na promoção da literacia para a saúde (LS), no apoio contínuo aos pacientes, e na vigilância de práticas seguras e ambientalmente responsáveis^(16-18)^.

Dessa forma, o artigo, ora apresentado, oferece caminhos para compreensão dos elementos que afetam a percepção de risco e a gestão de resíduos entre usuários e cuidadores envolvidos com a DP domiciliar. Além disso, contribui para o desenvolvimento de ações educativas e comunicativas voltadas à segurança do paciente e sustentabilidade ambiental.

## OBJETIVOS

Identificar as percepções de risco de profissionais, pacientes e cuidadores/familiares sobre o descarte adequado dos resíduos da DP domiciliar, com vistas à verificação de conteúdos/temas a serem considerados na elaboração de material audiovisual destinado a pacientes e profissionais de saúde.

## MÉTODOS

### Aspectos éticos

O estudo foi conduzido conforme diretrizes éticas nacionais e internacionais, e aprovado pelo Comitê de Ética em Pesquisa da Escola Politécnica de Saúde Joaquim Venâncio, cujo parecer está anexado à presente submissão. Todos os participantes foram informados sobre os objetivos, riscos e benefícios da pesquisa, bem como da liberdade de desistência, sem prejuízo ao tratamento. O Registro de Consentimento Livre e Esclarecido foi assinado presencialmente e/ou preenchido via formulário eletrônico (*Google Forms* ^®^), com cópia enviada aos participantes. O consentimento foi reafirmado verbalmente no início da aplicação em cada técnica de pesquisa. A confidencialidade foi garantida por meio do uso de codinomes na apresentação dos resultados do estudo.

### Tipo de estudo

Trata-se de estudo qualitativo, exploratório, guiado pelas diretrizes do *COnsolidated criteria for REporting Qualitative research*.

### Procedimentos metodológicos

Os procedimentos metodológicos se concentraram nas experiências e percepções de pacientes, cuidadores e profissionais em relação ao descarte de resíduos e à comunicação de risco no contexto da DP domiciliar. Foram utilizados como técnicas de coleta de dados entrevistas semiestruturadas e grupo focal (GF) *online*.

### Cenário do estudo

O estudo foi realizado em uma unidade de saúde privada conveniada ao Sistema Único de Saúde (SUS), localizada em Nova Iguaçu, Baixada Fluminense, Rio de Janeiro, que acompanha cerca de 350 usuários em tratamento renal substitutivo, sendo a única na região a oferecer DP. Contudo, esse tratamento não é disponibilizado por meio do SUS, devido ao baixo valor de reembolso, sendo realizado exclusivamente de forma privada. A escolha dessa unidade se deveu à sua importância no fluxo de referência e contrarreferência em níveis regional e municipal, ao número de pacientes atendidos, e à inserção profissional do primeiro autor na equipe de enfermagem da clínica.

### Fonte de dados

A seleção dos participantes ocorreu por amostragem não probabilística por conveniência, sendo recrutados dois grupos. Os critérios de inclusão para o primeiro grupo foram idade mínima de 18 anos, tempo mínimo de três meses em DP domiciliar e/ou no acompanhamento de pacientes em DP, condições clínicas e cognitivas preservadas. No primeiro grupo, dos 30 pacientes elegíveis para participar do estudo, 13 foram excluídos pelas seguintes razões: sete por estarem em treinamento para o uso da DP; três por se ausentarem da consulta; dois por estarem hospitalizados; e um pela ausência do cuidador responsável. Além dos pacientes, nesse primeiro grupo, também foram incluídos dez cuidadores por serem os responsáveis diretos pela execução da terapia no domicílio, após serem previamente treinados pela equipe de enfermagem.

O segundo grupo reuniu oito participantes convidados, por e-mail e *WhatsApp* ^®^, para compor o GF *online*. Os participantes foram selecionados por conveniência e através da rede de contatos dos autores. Na composição da amostra, considerou-se o envolvimento com a tema do estudo. Desse modo, foram convidados profissionais com experiência nas seguintes áreas: nefrologia (enfermeira); gestão de resíduos (arquiteto e enfermeira); LS (médica e assistente social); e comunicação (cineasta, especialista em comunicação de risco e jornalista/radialista, sendo este último também paciente em DP).

### Coleta e organização dos dados

O primeiro grupo participou das entrevistas semiestruturadas, realizadas em julho de 2024, nas instalações da clínica, em sala reservada e em horário escolhido pelos entrevistados. As entrevistas foram conduzidas pelo primeiro autor, com gravação autorizada e posteriormente transcritas. As entrevistas tiveram como objetivo identificar as percepções de risco relacionadas ao descarte de resíduos da DP em domicílio, a partir das vivências de pacientes e cuidadores diretamente envolvidos na prática cotidiana do tratamento. A opção pela abordagem qualitativa foi justificada pela sua capacidade de captar, com riqueza de detalhes, os significados atribuídos pelos participantes às suas experiências^(19,20)^.

As entrevistas foram aplicadas seguindo um roteiro com perguntas abertas previamente elaborado pelos dois primeiros autores, a partir do qual foram identificados os dados pessoais (nome, situação de trabalho/ocupação, escolaridade/formação, idade, estado civil), o conhecimento sobre a doença e seu tratamento com a DP (momento do diagnóstico, procedimentos do cuidado domiciliar), e a percepção sobre os resíduos gerados pela DP domiciliar e os riscos relacionados a eles. O roteiro foi adaptado à dinâmica de cada entrevista, totalizando 102,27 minutos de gravação (média de 6,04 minutos por sessão).

Com efeito, a partir do diálogo entre pesquisador e participante, foi possível acessar dimensões subjetivas e contextuais do cuidado domiciliar, revelando saberes, dúvidas, estratégias empíricas e lacunas informativas que dificilmente seriam evidenciadas por métodos quantitativos^(21,22)^. Mais do que dados objetivos, as entrevistas possibilitaram a compreensão de como os sujeitos interpretam os riscos, manejam os resíduos e o que consideram essencial comunicar em um material educativo. Portanto, os dados obtidos nessa etapa subsidiaram a elaboração do roteiro do GF, bem como os temas contidos no roteiro do audiovisual sobre descarte de resíduos da DP domiciliar. Assim, o GF se configurou como a etapa seguinte da investigação, voltada à validação e ao aprofundamento coletivo desses achados, possibilitando o confronto de percepções e a construção compartilhada do material educativo.

O GF foi agendado por meio da ferramenta Doodle^®^, e o encontro ocorreu em novembro de 2024, via plataforma *Microsoft Teams* ^®^, com duração de 171 minutos. O GF foi coordenado pelo primeiro e quarto autores, que observaram as interações entre os participantes, e mediaram o debate, em consonância com a proposta descrita por Alves para GF *online* ^(23)^. Inicialmente, o coordenador conduziu as apresentações e explicitou o objetivo do GF, que foi construir um roteiro com conteúdos relevantes para produção de um material audiovisual sobre descarte seguro dos resíduos gerados pela DP em domicílio. Em seguida, recorrendo a um roteiro com perguntas abertas, previamente elaborado pelos autores, foram discutidos os conhecimentos e percepções relacionados à DRC, ao tratamento domiciliar de DP e aos resíduos gerados nesse tipo de tratamento, bem como os riscos associados (Quadro 1).

**Quadro 1:** Grupo focal com especialistas e paciente: dinâmica e perguntas, Rio de Janeiro, Brasil, 2025

1º momento - Apresentação e informações gerais do grupo focal				

	Apresentação das questões éticas		Registro de Consentimento Livre e Esclarecido	

	Orientações sobre as regras do grupo focal		Será conduzido por três mediadores; Será gravado em imagem e áudio, mantendo a preservação da identidade dos participantes; Terá duração média de 90 a 140 minutos, com intervalos de dez minutos; O debate será disparado com perguntas abertas relacionadas à temática do estudo; As discussões poderão ser realizadas em texto e áudio, a critério de cada participante; Os participantes poderão inserir “emoticons” para sinalizar expressões, sentimentos e emoções vivenciadas, como também a pedido do moderador. Antes de finalizar o debate de cada pergunta, os participantes serão convidados a inserir “emoticons”, representando sua expressão diante do assunto discutido.	

**2º momento - Debate**				

	Perguntas sobre a experiência dos participantes sobre a doença renal crônica e a diálise peritoneal		Qual seu conhecimento sobre a doença renal crônica? Possuem alguma experiência sobre a diálise peritoneal? Sabem como é realizada?	

	Perguntas sobre o descarte dos resíduos da diálise peritoneal		Vocês conhecem quais resíduos são gerados? Já se questionaram sobre o descarte dos resíduos da diálise peritoneal em casa? Como podemos descartar esses resíduos de forma segura?	

	Construção do roteiro para o audiovisual		Exposição e análise de materiais instrucionais existentes para os pacientes e familiares em diálise peritoneal; O que precisamos para comunicar o descarte de forma segura dos resíduos da diálise peritoneal voltados aos pacientes, familiares e cuidadores? O meio de divulgação (audiovisual) é o mais adequado? Quais informações poderão ser incluídas? Quais dificuldades existem? Quais soluções podemos ter	


### Análise dos dados

A análise das entrevistas e do GF foi realizada por meio de abordagem mista, com análise quantitativa descritiva das variáveis sociodemográficas e análise qualitativa fundamentada na técnica de análise de conteúdo temática^(24)^. A análise de conteúdo foi desenvolvida em três etapas. Na primeira etapa, pré-análise, foram realizadas as transcrições integrais das entrevistas e do GF e a leitura flutuante das informações transcritas, identificando as informações, as frequências com que as palavras se repetem e os temas iniciais. Observou-se cada texto de maneira isolada e transversalmente, elencando os elementos de significação a partir dos temas iniciais. Nessa etapa, foram selecionados temas iniciais que deram origem aos subgrupos temáticos. Alguns desses temas surgiram de forma inesperada, a partir das falas dos participantes, enquanto outros derivaram diretamente das perguntas formuladas nos roteiros das entrevistas e do GF.

A segunda etapa, exploração do material, contemplou o processo de codificação dos temas iniciais, levando-se em conta os seleção de palavras, frases e parágrafos que geraram as unidades de registros, bem como as regras definidas para contagem, classificação e agrupamento das informações em categorias temáticas, tendo como base a convergência semântica^(24,25)^. Desse modo, os textos das entrevistas e do GF foram agrupados em temas iniciais, unidades de registro, e, posteriormente, agrupadas em unidades temáticas.

Na terceira etapa, inferência e interpretação, foi realizado o tratamento dos resultados da etapa de codificação, levando-se em conta a análise das falas a partir dos critérios de exaustividade, representatividade, homogeneidade e pertinência. Esse procedimento visou à identificação dos conteúdos, expressos e latentes, existentes no material analisado. Na sequência, esses dados foram reagrupados através de categorias analíticas e suas respectivas subcategorias, possibilitando interpretações consistentes em relação ao objeto do estudo. As falas citadas nos resultados estão identificadas pelas letras “P” (paciente) e “C” (cuidador), seguidas do número de inserção, sexo e idade (P:01, mulher, 30 anos). O paciente radialista, que também participou do GF, foi identificado pela sigla “PR”. Os especialistas foram identificados pela letra “E”, acompanhada das iniciais da área de atuação, como EN (nefrologia), ELS (literacia para a saúde), ESA (saúde ambiental), ER (resíduos), ECR (comunicação de risco) e EC (cineasta).

## RESULTADOS

Participaram das entrevistas 17 pessoas (sete pacientes e dez cuidadores), envolvidas diretamente na DP domiciliar, com idade entre 18 e 89 anos. A maioria dos pacientes tinha idade acima de 40 anos, era casada, e com escolaridade entre o ensino fundamental e o superior completo. Entre os cuidadores, houve predominância do sexo feminino, faixa etária entre 40 e 69 anos, maior nível de escolaridade e de inserção no mercado de trabalho. Quanto ao tratamento, cerca de dois terços dos pacientes utilizavam DP automatizada, e a maioria deles estava em terapia há menos de três anos. A maioria dos pacientes e cuidadores residia em municípios da Baixada Fluminense e Região Metropolitana do Rio de Janeiro (Tabela 1).

**Tabela 1:** Características sociodemográficas e clínicas segundo tipo de participante (paciente ou cuidador), Rio de Janeiro, Brasil, 2025

Variável	Paciente		Cuidador	
	n	%	n	%
**Sexo**				
Masculino	3	42,9%	**4**	**40%**
Feminino	4	57,1%	6	60%
**Faixa etária (anos)**				
18-29	2	28,6%	-	-
30-39	-	-	2	20%
40-69	4	57,1%	7	70%
70-89	1	14,3%	1	10%
**Estado civil**				
Solteiro	1	14,3%	1	10%
Casado	5	71,4%	9	90%
União estável	-	-	-	-
Divorciado	1	14,3%	-	-
Viúvo	-	-	-	-
**Escolaridade**				
Analfabeto	-	-	-	-
Ensino fundamental incompleto	1	14,3%	-	-
Ensino fundamental completo	1	14,3%	1	10%
Ensino médio incompleto	1	14,3%	-	-
Ensino médio completo	2	28,6%	4	40%
Ensino superior incompleto	-	-	2	20%
Ensino superior completo	2	28,6%	3	30%
**Atividade profissional**				
Estudante	-	-	-	-
Ensino	1	14,3%	1	10%
Indústria/comércio	1	14,3%	2	20%
Trabalho informal	1	14,3%	2	20%
Profissional liberal	-	-	2	20%
Atividade doméstica	2	28,6%	2	20%
Aposentado	1	14,3%	-	-
Funcionário público	-	-	-	-
Sem atividade	1	14,3%	2	20%
**Modalidade de diálise**				
Diálise peritoneal automatizada	11	64,7%	-	-
Diálise peritoneal ambulatorial contínua	6	35,3%	-	-
**Tempo de tratamento (anos)**				
**Até três anos**	14	82,4%	-	-
> três e < cinco anos	2	11,8%	-	-
> cinco anos	1	5,9%	-	-

O GF *online* contou com a participação de um paciente e sete profissionais com graduação em diversas áreas (enfermagem, medicina, serviço social, arquitetura, comunicação e produção audiovisual), sendo cinco deles doutores. Houve predominância feminina, sendo a maioria dos participantes residente no estado do Rio de Janeiro.

## DISCUSSÃO

Na análise de conteúdo das entrevistas e do GF, descritas no Quadro 2, foram identificados 37 temas iniciais, reagrupados em quatro subcategorias: a) Experiências e saberes sobre a doença renal crônica e a diálise peritoneal; b) Práticas e cuidados referentes à diálise peritoneal domiciliar; c) Resíduos gerados na diálise peritoneal domiciliar: descarte seguro; d) Comunicação e saúde: material audiovisual sobre a diálise peritoneal domiciliar. Por fim, essas quatro subcategorias foram reagrupadas em duas categorias de análise, que sintetizam as interpretações e inferências à luz da literatura especializada, quais sejam “Do cuidado ao resíduo” e “Estratégia de comunicação utilizada pelo profissional de saúde na diálise peritoneal”.

**Quadro 2:** Categorias, subcategorias e principais inferências da análise temática, Rio de Janeiro, Brasil, 2025

Categoria	Subcategoria	Temas iniciais
**Do cuidado ao resíduo**	Experiências e saberes sobre a doença renal crônica e a diálise peritoneal	- Doença renal crônica: experiência, saberes e sentimentos - Diálise peritoneal: experiência, sentimentos, mitos e verdades - Vulnerabilidade social de pessoas em diálise peritoneal no Sistema Único de Saúde - Aspectos socioculturais relacionados à diálise peritoneal (impacto na vida social, rotina diária, aceitação e adaptação ao tratamento) - Rede de apoio familiar no enfrentamento da doença
	Práticas e cuidados referentes à diálise peritoneal domiciliar	- Papel do enfermeiro no treinamento do paciente/família/cuidador - Técnica da diálise peritoneal/preparo do material - Consciência em realizar a técnica correta - Facilidade de aplicação - Rigorosidade na higienização - Materiais utilizados na diálise peritoneal
	Resíduos gerados na diálise peritoneal domiciliar: descarte seguro	- Tipo de resíduo gerado pela diálise peritoneal - Desinformação sobre o descarte dos resíduos da diálise peritoneal em domicílio - Insegurança em manipular os resíduos gerados pela diálise peritoneal - Preocupação pelos trabalhadores que manuseiam os resíduos descartados - Reflexão das desigualdades sociais em cada região do Brasil em relação ao descarte de resíduos - Políticas públicas e resíduos - Resíduo doméstico é inconveniente - Cuidados com os resíduos no ambiente domiciliar - Benefícios do descarte adequado para as pessoas e para o meio ambiente - Descarte com conforto - Solução a curto prazo: recolha dos materiais pela transportadora - Solução a médio e longo prazo: logística reversa.
**Estratégia de comunicação utilizada pelo profissional de saúde na diálise peritoneal**	Comunicação e saúde: material audiovisual sobre a diálise peritoneal domiciliar	- Comunicação entre profissionais de saúde e pacientes/ cuidadores - Utilização de linguagem simples - Perspectiva da salutogênese - Estratégias comunicacionais: ambientação do tema; ponto virada; mensagens positivas: mostrar empatia no material; mensagens chave; público destinado; canais e formatos; percursos a ser utilizado - Personagem realista: enfermeiro e paciente na composição do roteiro

### Do cuidado ao resíduo

A categoria “Do cuidado ao resíduo” compreende três subcategorias que abordam a experiência de adoecer, o cotidiano do tratamento domiciliar com DP, e os desafios relacionados ao cuidado, ao manejo dos resíduos e aos riscos associados à gestão dos resíduos gerados.

A primeira subcategoria, “Experiências e saberes sobre a doença renal crônica e a diálise peritoneal”, sinaliza a descoberta tardia e o pouco conhecimento e/ou desconhecimento sobre a doença renal e DP. Os relatos de pacientes e cuidadores apontam que o diagnóstico da DRC frequentemente ocorreu de forma inesperada, após episódios graves de adoecimento. A experiência do processo de adoecer é única, e o início da terapia é vivenciado como um momento de perda e luto simbólico.

*Paralisei minha vida por causa da diálise.* (P1, homem, 23 anos)

*Você ter uma doença que te limita, né? Você não tem mais aquela vida que você já teve um dia, né? De poder ter uma liberdade total em todos os sentidos da vida.* (P8, homem, 49 anos)

A carga emocional e o significado da doença impactam o corpo, geram problemas psicológicos, e impõem dependência de acompanhamento especializado e alterações na rotina familiar e social. A decisão pela terapia se mostrou mais viável com apoio profissional e familiar, embora a DP ainda seja pouco difundida.

*No começo, eu era regrada, eu fazia tudo direitinho. E depois, eu fui meio que me abandonando. Aí também é problema psicológico* [...] *a gente se priva muito para viver.* (P14, mulher, 29 anos)

*O enfermeiro me apresentou essa nova possibilidade... aquilo já me deu uma certa tranquilidade.* (PR)

*Eu tenho a ajuda do meu pai, que é muito importante* […] *acho que é 50% do tratamento.* (PR)

A dificuldade de acesso à DP e a desinformação contribuem para mitos sobre critérios de adesão como exigências rígidas sobre o domicílio.

*Quando eu conheci esse tratamento, as pessoas falavam para mim ter um quarto de hospital em casa.* (PR)

*Existia um conceito de que, para fazer diálise peritoneal, tinha que ter um quarto só, sem cachorro, banheiro exclusivo. Esse conceito mudou, inclusive porque a diálise peritoneal atende pessoas que moram distante.* (EN)

A subcategoria “Práticas e cuidados referentes à diálise peritoneal domiciliar” evidencia a importância do treinamento conduzido pelo enfermeiro no preparo do paciente, família e cuidador, favorecendo a apropriação das técnicas requeridas e a segurança no manejo domiciliar. Os participantes relataram que a rotina inclui cuidados rigorosos de higiene, preparo do ambiente e manuseio correto dos insumos.

*Primeiro, a gente tem que limpar o ambiente todo, né? O quarto. Limpo tudo. Passo álcool e cloro em tudo. Espero 30 minutos. Aí depois eu lavo os aparelhos, com sabão, lavo as mãos, bem lavado, passo álcool e conecto. Depois de conectado, tiro o líquido, né? E termino. E faço a vida normal”.* (P17, mulher, 52 anos)

Um aspecto que chamou atenção de pacientes e cuidadores é o volume de insumos entregues mensalmente nas residências, acondicionados em caixas de papelão protegidos por invólucros plásticos. Além disso, em relação ao tratamento, foi destacada a necessidade de usar materiais para higienização e segurança do procedimento.

*Chegaram aqui em casa as caixas com o peritosteril, que é o líquido que a gente usa na diálise peritoneal, e caixas com os equipos* [...] *que é a maneira que a gente se conecta na máquina, na bolsa, no meu cateter. E aí, a cada diálise, eu uso um pacote.* (PR)

*Tem o álcool 70, para higienizar as mãos, a máscara, para evitar e minimizar qualquer risco de infecção. O curativo, a higienização, usando soro, gaze* [...] *a questão da agulha é um pouco relativo, porque, às vezes, aparecem algumas fibrinas que acaba travando a passagem do líquido no cateter, e aí a gente injeta na bolsa uma solução, mas não necessariamente esse processo da agulha tem que ser feito todos os dias.* (PR)

A terceira subcategoria, “Resíduos gerados na diálise peritoneal domiciliar: descarte seguro”, revela lacunas de informação, insegurança no manejo dos resíduos e preocupação com riscos à saúde pública e ao meio ambiente. Embora alguns descrevam suas rotinas de descarte, muitos não têm clareza sobre qual é o procedimento adequado.

*Pego o material, né? Corto aquele líquido... jogo fora o líquido no tanque* [...] *pra poder ir pro esgoto* [...] *e jogo fora o material.* (P17, mulher, 52 anos)

*Aí depois que termina, a gente também coloca a máscara, retira ele do equipamento. Passa álcool nas coisas e descarta o material.* (C16, mulher, 35 anos)

Houve relatos que expressaram a incerteza sobre os riscos e uma preocupação recorrente com a exposição dos trabalhadores da coleta.

*Aí a gente, né* [...] *bota num saco separado para o lixeiro levar* [...] *com medo de contaminação mesmo.* (P17, mulher, 52 anos)

*O risco não é só para o paciente; é para todos que envolvem*. (C2, mulher, 43 anos)

As falas reforçam uma ausência de informações sobre essa etapa do tratamento. Isso é agravado pela diversidade de materiais utilizados, que dificulta a categorização quanto ao risco e, consequentemente, o descarte correto.

*Existe um vácuo de informações quando chega nessa parte do descarte. Não tem ainda um manual muito bem específico. Aquela bolsa plástica que tem resíduos líquidos ali dentro, as borrachas do equipo, a tampinha do cateter, vai jogar no lixo? Não tem que ter um preparo?* (PR)

O descarte de perfurocortantes e o acúmulo de resíduos configuram um desafio que também geram dúvidas.

*É, essa agulha, justamente nossa dúvida, eu junto essas agulhas e levo para o enfermeiro descartar na clínica.* (PR)

*Eu tenho trocentas caixas que eu não consigo dar destino, porque o lixeiro não quer levar, e queimar fica muito difícil, ninguém quer, não está sendo vendido mais, ninguém quer porque é baixo, mas então se torna um peso.* (C13, homem, 58 anos)

Os especialistas apontaram falhas na legislação, ressaltando que, no ambiente doméstico, os resíduos podem gerar riscos e incômodos, e, em locais sem infraestrutura sanitária adequada, esses riscos se tornam ainda mais graves.

*A gente tem uma legislação ambiental muito bem colocada, mas existem pontos ainda falhos nessa questão do descarte* [...] *e dentro dessa questão, a gente tem a classificação desses resíduos.* (ESA)

*Resíduo é escala, quanto maior a escala, maior o problema. No ambiente doméstico, ele impõe riscos ou inconvenientes: odor, atração de vetores* [...] *a relação com o resíduo é que determina o que ele vai fazer com aquilo.* (ER)

*Hoje temos duas empresas no Brasil que fornecem material de diálise peritoneal: uma descarta o efluente direto no esgoto, a outra, não. São bolsas onde esse efluente fica e vai ser drenado normalmente no vaso sanitário.* (EN)

A destinação inadequada do efluente em locais sem saneamento, como fossas próximas a poços, aumenta os riscos de contaminação.

*Temos situações onde pacientes usam água de poço e destinam o efluente sanitário numa fossa que está a 10 ou 15 metros daquele poço* [...] *quando ele joga aquilo dentro de uma fossa e capta água do lençol freático, ele pode estar se sabotando.* (ER)

Como soluções, os especialistas destacaram a logística reversa e a recolha de materiais pela transportadora, além do uso de recipientes rígidos para perfurocortantes.

*Legalmente, o gerador também tem responsabilidade no tratamento. O fabricante vai ter que recolher de alguma forma.* (ER)

*Quem entrega o material poderia recolher papelão e plástico reciclável, não as bolsas com resíduo de diálise.* (EN)

*No médio e no longo prazo, aplicar a logística reversa. A curto prazo, criar micrologística onde o entregador recolhe periodicamente materiais específicos.* (ER)

*Eu oriento juntar agulhas e seringas em frascos rígidos e largos, para que sejam descartados da forma mais correta possível.* (ESA)

*Na maioria dos municípios, não existe coleta separada de resíduos.* (ER)

### Estratégia de comunicação utilizada pelo profissional de saúde na diálise peritoneal

A categoria “Estratégia de comunicação utilizada pelo profissional de saúde na diálise peritoneal”, derivada da subcategoria “Comunicação e saúde: material audiovisual sobre a diálise peritoneal domiciliar”, reúne reflexões de diferentes profissionais e pacientes, destacando elementos fundamentais para subsidiar a elaboração do roteiro do audiovisual educativo sobre o descarte de resíduos da DP.

Os participantes endossaram o formato de audiovisual indicando seu potencial de interatividade, recomendando-se a inclusão de “*QR Code* e outros *links* com informações hierarquizadas” (ECR). Igualmente, propuserem vídeos curtos e objetivos, com linguagem direta e motivacional, de acordo com perfil do público e o canal de divulgação. Com enfoque da LS, discutiu-se a importância de hierarquizar os conteúdos retratando o cotidiano do tratamento - recebimento dos insumos, preparo para o procedimento da DP, o manejo dos resíduos e o descarte seguro. Para isso, os participantes sugeriram que os personagens fossem pacientes, profissionais e cuidadores envolvidos no cuidado, valorizando-se a humanização do cuidado, a autonomia, a segurança do paciente e a qualidade de vida. Na composição dessa narrativa, também foi recomendado abordar a interação entre profissional de saúde paciente e cuidador, enfatizando o vínculo, a escuta ativa e a empatia. A comunicação, segundo eles, é uma via de mão dupla, promovendo a corresponsabilidade no cuidado, pois, de acordo com eles:

*Não adianta só dizer o que é certo. Tem que ouvir, entender as dificuldades.* (EC)

*Você precisa primeiro falar a linguagem que o público vai entender. Não adianta falar a linguagem médica.* (PR)

Aliada a essa perspectiva, a comunicação de risco foi apontada como estratégica para segurança do paciente, sendo indicada a construção de uma narrativa acessível que motive a ação, associando o descarte correto ao bem-estar individual e coletivo. Os participantes retiraram a necessidade de contextualizar o descarte dos resíduos dentro do processo completo da terapia, considerando que “não tem como entender o descarte sem entender todo o processo que o antecede” (EC).

A partir desses elementos, propõe-se um pré-roteiro de um audiovisual sobre DP domiciliar e descarte de seguro, com foco na promoção de saúde e empoderamento dos sujeitos envolvidos. O pré-roteiro contempla os seguintes aspectos: ambientação do tema; definição de mensagens-chave; clareza conceitual; uso de narrativas motivacionais; adaptação do formato ao canal de veiculação; estímulo ao autocuidado; e valorização do vínculo entre profissional e paciente. O Quadro 3 descreve esses aspectos.

**Quadro 3:** Proposta para o roteiro do audiovisual, Rio de Janeiro, Brasil, 2025

Título: “Diálise peritoneal: cuidar de si e do mundo”
**PÚBLICO-ALVO:** Pacientes em diálise peritoneal. Familiares e cuidadores formais e informais. Profissionais de saúde que atuam na educação em saúde domiciliar.
**AÇÃO E PARTICIPANTES** Formato principal: Conversa realista entre enfermeiro e paciente, simulando um momento de orientação domiciliar. Cenário: ambiente doméstico adaptado ou espaço de atendimento acolhedor. Participantes: Enfermeiro: postura empática, clara, cuidadosa. Paciente: representa as dúvidas e sentimentos comuns. (Opcional) Familiar ou cuidador presente como apoio e ouvinte ativo.
**ESTRATÉGIAS COMUNICACIONAIS** Linguagem simples e direta. Uso de conceitos da literacia para a saúde - explicações visuais, comparações simples e exemplos práticos. Comunicação de risco (ex.: riscos de infecção, descarte incorreto). Perspectiva salutogênica: foco na autonomia, bem-estar e aspectos do cotidiano de paciente e cuidadores. Ênfase nos aspectos ambientais: consciência ecológica no descarte dos resíduos.
**FORMATO** **Opção 1 - Vídeo único** Duração: aproximadamente dez minutos. Formato compacto, ideal para apresentação em ambulatórios, sessões de acolhimento ou como conteúdo digital avulso. **Opção 2 - Série (minissérie educativa)** Três episódios com duração de aproximadamente dez minutos totais. Permite aprofundamento gradual de temas, maior vínculo com os personagens e reforço da aprendizagem. Permite seu uso seriado ou não, podendo servir para diferentes propósitos e públicos.

## DISCUSSÃO

O desconhecimento sobre os fatores que levam à DRC é amplamente documentado^(26)^. Por ser assintomática e de evolução lenta, geralmente é diagnosticada tardiamente, quando já há necessidade de diálise^(27)^. A falta de informação, possivelmente decorrente de comunicação insuficiente dos profissionais de saúde, afeta de maneira desigual os diferentes grupos populacionais. A escassez de estudos sobre o impacto da mídia e dos provedores na compreensão da doença dificulta a definição de estratégias comunicacionais mais efetivas^(28)^. A DRC impacta a vida dos pacientes, levando frequentemente ao abandono do emprego e à redução das atividades sociais. A progressão da doença provoca declínio físico e psicossocial, exigindo acompanhamento contínuo, comprometendo a autonomia e aumentando a dependência de terceiros^(29,30)^. As demandas do tratamento interrompem planos de vida, gerando sentimentos de limitação e perda de controle^(30)^.

A DP emerge como alternativa que promove autonomia por meio do tratamento domiciliar, embora seja pouco utilizada devido às limitações estruturais, financeiras e à falta de conhecimento dos pacientes, agravada por informações inadequadas das equipes de saúde^(26)^. Nesse contexto, enfermeiros e médicos nefrologistas exercem papel central ao orientar pacientes, estimulando participação nas decisões e adesão ao tratamento^(31)^.

No Brasil, marcado por desigualdades regionais, culturais e socioeconômicas, pessoas em moradias sem condições adequadas para realização de terapias domiciliares como a DP se tornam mais vulneráveis. A ausência de infraestrutura mínima, aliada a baixos níveis de escolaridade e renda familiar, compromete a eficácia do tratamento e aumenta os riscos de complicações, inclusive relacionados ao manejo inadequado de resíduos de saúde em domicílio^(32)^. O suporte familiar é fundamental para enfrentar as demandas da DRC, fornecendo apoio emocional, social e financeiro, favorecendo adesão e qualidade de vida^(33)^.

O êxito da DP depende de ambiente adequado, treinamento contínuo e acompanhamento regular. O enfermeiro conduz a capacitação de pacientes, familiares e cuidadores, assegurando domínio técnico, execução correta do procedimento e autonomia no manejo domiciliar. Além disso, orienta sobre o manejo seguro e responsável dos resíduos gerados^(34)^. No entanto, a DP gera diversos tipos de resíduos plásticos, tais como o policloreto de vinila e polipropileno, perfurocortantes, ampolas de vidro, soluções dialíticas, e papel/papelão, com volumes significativos anuais^(13)^. A adoção de estratégias sustentáveis pelas empresas prestadoras de serviços e unidades de saúde, como reciclagem, logística reversa e práticas de “*Green Nephrology*”, pode reduzir a pegada ambiental, promovendo reaproveitamento da água e uso de materiais recicláveis^(12,35)^.

O manejo inadequado de resíduos representa riscos ocupacionais, ambientais e à saúde pública. Perfurocortantes e ampolas podem causar acidentes e o descarte incorreto de medicamentos contribui para resistência bacteriana^(36,37)^. Além disso, técnicas de higiene insuficiente durante a DP aumentam o risco de peritonite e transferência precoce para HD^(38,39)^.

No Brasil, práticas de descarte domiciliar refletem desigualdades regionais e socioeconômicas, sendo frequente o descarte inadequado de seringas, agulhas e plásticos^(32)^. Apesar das normas da Resolução da Diretoria Colegiada nº 222, de 28 de março de 2018, da Agência Nacional de Vigilância Sanitária, muitos resíduos ainda são destinados ao lixo comum, elevando riscos sanitários e ambientais^(32,40)^.

Insumos devem ser armazenados em locais secos, limpos e inspecionados quanto à integridade das embalagens^(41)^. A técnica asséptica, com ênfase na higiene das mãos, permanece medida preventiva essencial^(38,39)^. A logística reversa e a reciclagem são estratégias promissoras, embora enfrentem desafios técnicos e financeiros, enquanto a incineração, apesar da eficiência, exige alto controle ambiental e investimento^(12,36)^.

A comunicação entre profissionais, pacientes e cuidadores é essencial para compreensão, adesão e bem-estar. Linguagem simples, direta e acessível aumenta eficiência, reduz ambiguidades e fortalece a segurança^(42)^. A perspectiva salutogênica orienta a comunicação, enfatizando fatores que promovem saúde e fortalecem o sentido de coerência, melhorando capacidade de lidar com adversidades^(43)^.

Estratégias comunicacionais estruturadas, com ambientação do tema, mensagens-chave, exemplos práticos, reforço positivo e inclusão de personagens realistas, como enfermeiros e pacientes, facilitam identificação do público e promovem maior adesão e compreensão^(42)^. Canais e formatos digitais, alinhados ao público-alvo e valores institucionais, potencializam a eficácia^(44)^. Formação contínua e especializações aprimoram habilidades, satisfação profissional e desfechos clínicos^(45)^.

Em síntese, a integração da salutogênese, com estratégias comunicacionais planejadas, fortalece autonomia e promove cuidado humanizado, segurança e bem-estar, sendo base para materiais educativos acessíveis, acolhedores e informativos direcionados à prática cotidiana, prevenção de riscos e cuidado ambiental.

### Limitações do estudo

Entre as principais limitações deste estudo, destacam-se a amostragem por conveniência, que pode ter introduzido viés de seleção e restringido a representatividade dos diferentes perfis de pacientes e cuidadores em DP, e a realização do GF *online*, que pode ter limitado a observação de elementos não verbais, como expressões faciais e gestos, importantes para análise qualitativa. Além disso, embora tenha sido elaborado um pré-roteiro para material audiovisual educativo, o vídeo como produto final ainda será desenvolvido e validado com o público escolhido.

### Contribuições para as áreas da enfermagem, saúde ou políticas públicas

O estudo reforça a relevância do papel da enfermagem no cuidado ao paciente de DP e na educação em saúde, principalmente diante da necessidade de fundamentar a percepção de risco com base em conhecimentos técnicos, desenvolvendo a LS entre pacientes e cuidadores. Os resultados deste estudo buscaram subsidiar a produção de um roteiro para um audiovisual contextualizado que contribua para qualificar a atuação do enfermeiro, pacientes e cuidadores envolvidos na promoção de práticas de cuidado domiciliar seguras e ambientalmente responsáveis.

## CONSIDERAÇÕES FINAIS

O estudo atingiu seu objetivo, ao identificar conteúdos-chave sobre a percepção de risco no descarte de resíduos da DP domiciliar, a partir de relatos de pacientes, cuidadores e profissionais. Os resultados evidenciaram dúvidas sobre o descarte adequado, lacunas na legislação brasileira e a relevância de práticas ambientalmente seguras, como coleta seletiva e logística reversa.

As contribuições dos participantes destacaram a necessidade de estratégias educativas acessíveis, com ênfase em comunicação de risco e LS como ferramentas para promover autonomia, senso crítico e práticas seguras. O pré-roteiro de material audiovisual educativo, elaborado a partir dessas informações, surge como resposta concreta às lacunas identificadas, com potencial para apoiar o cuidado domiciliar, capacitar profissionais e ampliar a discussão sobre sustentabilidade na terapia renal.

## Data Availability

Os dados de pesquisa só estão disponíveis mediante solicitação.
